# Bronzed Skin and Disease of the Supra-Renal Capsules

**Published:** 1856-05

**Authors:** 


*Bronzed Skin and Disease of the Supra-Renal Capsules.*

We extract the following remarks from Mr. Hutchinson's paper (London Med. Times and Gaz.) upon Bronzed Skin, based upon an analysis of 27 cases of the affection, all that have hitherto been brought to his notice. It will be remembered that Dr Addison first drew the attention of the profession to this condition, in connexion with organic disease of the supra-renal capsules :—

*Symptoms attending Diseases of the Supra-Renal Capsules.*

1. *Change of color of the skin.*—The term "bronzing" probably conveys as good an idea of the character of the appearance assumed by the skin in this disease as could well be given. It resembles stikingly the color of a bronzed statue from which the gloss has been rubbed off. Pressure has no effect in causing its diminution. It seems as a rule to commence first in patches with ill-defined borders, on those parts most exposed to the sun and to friction, the neck, the back of the hands, the fronts of the thighs, the arms, etc. Around the nipple, and in other parts where pigment naturally abounds, it is generally well-marked, while others, possessing little or no pigment originally, as the palms of the hands, the ungual matrices, etc., remain as pale as ever. This tendency to show itself in patches, is strongly in support of the belief that the change is really one of deposit of pigment; which derives further confirmation from the circumstance, that in not a few cases the punctate, or even patchy deposit of black matter, was observed in the mucous membrane of the mouth, and in the serous investment of the abdominal viscera. The conjunctiva usually remains pale and pearly, a condition which well distinguishes true bronzing from the various states of jaundice. The changed color of skin, although most important for purposes of diagnosis, is probably of very minor consequence among the other departures from health which attend renal capsular disease.

2. *Debility.*—Next to bronzing of the integument the extreme and peculiar feebleness manifested appears to be the most striking of the symptoms. Without any evidence of thoracic disease, without any great loss of flesh, the patient becomes liable to faintings, loses energy, is unable to exert either body or mind, and, in short, appears to be on the point of death from sheer weakness.

3. *Emanciation.*—Flabbiness of the solids rather than actual wasting is characteristic of the condition and seems true of the majority of cases.

4. *Anæmia.*—In almost all cases, there would seem to have been present great depravation of the colored constituents of the blood, as manifested by the pallor of those parts not involved in the bronzing, the general flabbiness of the muscles, the pearly state of the conjunctiva, etc.

5. *The Pulse.*—With a few exceptions, in which it became rapid, the pulse has generally been of but average frequency, and peculiar only in its extreme softness and compressibility.

6. *The tongue.*—It does not appear that any state of the tongue other than that common to most conditions of debility has been observed in connexion with this disease.

7. *Dyspepsia.*—In almost all cases prior to death, and in many for protracted periods, great irritability of the stomach was present. In most there was loss of appetite, more or less persistent nausea, and occasional vomiting, with pain and sense of sinking at the epigastrium. In the majority, it would seem that the bowels have been costive rather than otherwise, while, in a few, attacks of diarrhœa had occurred. In several instances the patients had been liable to "biliousness." Much more detailed observations as to the symptoms of indigestion present are desirable.

8. *The Urine.*—The urine was tested for albumen in many of the cases, and in some for sugar; but in no instance was any important departure from its normal constitution observed.

9. *Lumbar Pain.*—Aching, more or less severe, in the back or loins, was a symptom present in a considerable proportion of the cases. In two or three of these there was, however, disease of the vertebræ, by which it might have been occasioned; and the evidence respecting it is not such as to induce us to attach much importance to its signification.

10. *Nervous symptoms, convulsions, etc.*—Symptoms referable to disorder of the cerebro-spinal functions occurred in several of the cases. In three, epileptiform convulsions preceded death. In one, failure of memory, and remarkable change in temper, was observed; and in a second, numbness of the fingers, legs, and the tip of the tongue, had been present. In one, the man had suffered from tic douloureux.

11. *Odor of the Body*—In two cases, both under care at the Brighton Hospital, it was noticed that a peculiarly disagreeable odor was exhaled from the patient's body. In one this was present for a few weeks, and in the other only for a few days prior to death. The phenomenon is not mentioned in the reports of any other case in the series, and Dr. Addison informs us that it was not noticed in any case under his observation.

*Mode of Death.*—Speaking generally, we may say that the phenomena attending death are those of utter prostration of the vital powers, not unfrequently complicated by disturbance of the nervous functions.

*Diagnosis.*—The combination of a bronzed state of the skin with great systemic debility may be held indicative of disease of the supra-renal capsules; and the more marked these conditions are, the more positively may the opinion be formed. [Jaundice, browning from exposure to the sun, Pityriasis versicolor, and the diffused brown muddiness of some other cachexiæ, may be mistaken for bronzing—a little care, however, will serve to distinguish them.] In the meantime, it should be borne in mind that in all cases in which bronzing is to be held as positively indicative of diseased capsules there ought to be traces of patching and mottling in some parts; and that in proportion as the tint is equally diffused over the whole body is the diagnosis doubtful.

*Prognosis and Treatment.*—We have as yet no reason for believing that in cases of true bronzing of the skin, the Physician can do other than give the most unfavorable prognosis. No recovery has yet been recorded, nor, indeed, has even temporary improvement under treatment been very marked in any case. After such an avowal it may, perhaps, seem superfluous to speak of treatment, since in our search for principles to guide us in it, we can avail ourselves only of that very delusive light which pathology furnishes. There seems, however, reason for believing, that the morbid changes to which the supra-renal bodies are liable are most of them more or less closely allied to inflammation; and from this fact one might, perhaps, be justified in selecting remedies from the class of drugs known to possess influence over that process. The exhibition of a course of mercury (in very small doses), or the use of the iodide of potassium, the patient's strength being meanwhile supported by a nutritious but non-stimulating diet, would probably be the most rational practice which could be suggested for a case of bronzed skin. As the change in color is, however, only produced, in all probability, after the organic disease has considerably advanced, there would not, it is to be feared, be much to be hoped for in the way of restoration of function.

*Morbid Anatomy.*—The cases recorded show examples of the following conditions in the supra-renal bodies. A remarkable symmetry of disease appears to have existed in all excepting the cases of cancer.

1. Acute and recent inflammation ending in abscess (Case 17).

2. Atrophy with fibro-calcareous concretions. This condition appears to have been present in seven cases. In some of them cysts existed, and in several a fluid matter resembling pus was contained in the cysts, and bathed the solid fibro-calcareous concretions. These changes probably result from inflammation of chronic character. The complete disorganization of the viscus is usually effected, and in all the cases recorded both glands were involved (Cases 2, 4, 7, 12, 13, 14, and 16).

3. The conversion of the viscus into a sort of fibroid structure, with great enlargement and induration. This occurred in Cases 1 and 6, and in both all trace of healthy tissue was lost.

4. The deposit of tubercle. In three instances (Cases 3, 5, and 9,) masses of deposit resembling tubercle were observed, and, coincidently, great enlargement of the organ and loss of normal texture. In two the deposit existed in both organs, and in one there was no tubercle in other viscera. It may be doubted whether the deposit is not really more nearly allied to some form of fibrinous effusion, the result of inflammation, than to true tubercle.

5. *Cancer.*—In six cases (Cases 7, 8, 10, and 11), the deposit of cancer has been observed. In all it was secondary to the same disease in other organs. In four it affected but one organ, and in two both were involved. In but one was complete disorganization of both affected.

In the "Foreign Correspondence" of the same Journal is the following:

Three communications made last month, at the *Societé de Biologie*, by Dr. Brown-Séquard, seem to introduce some interesting particulars into our knowledge of the pathology of the supra-renal capsules. At first, Dr. Brown-Séquard mentioned that, more than four years ago, in 1851, (*Comptes Rendus de la Soc. de Biol.*, 1851, pp. 146, and *Gaz. Méd. de Paris*, 1852, pp. 73,) he had reported the results of many experiments, proving that, after certain injuries to the spinal cord in animals, the supra-renal capsules always become hypertrophied, after a few months. If the animals are killed a few days after the injury to the spinal cord, the supra-renal capsules are found in a state of hyperæmia, and sometimes they contain a clot of blood in their medullary substance. If the animal lives two or three months, or much more, after the injury, these organs are found to be twice or three times as large as in their normal state. The part which then becomes hypertrophied is the medullary substance. Since the year 1851, Dr. Brown Séquard has had the oportunity of opening more than a hundred animals, upon which he had severely injured the spinal cord, and, in all these cases, he has found the supra-renal capsules in a state of congestion when the injury was recent, and hypertrophied when it had been afflicted many months before.

These facts seem to establish positively the existence of an influence of the spinal cord on the blood-vessels, and on the nutrition of the supra-renal capsules.

In the ensuing No. of the same it is stated:

In a recent communication to the *Societe de Biologié*, Dr. Brown-Séquard has almost completed the exposition of the results of his researches upon the physiology and pathology of the supra renal capsules. He has found that the extirpation of the two capsules in dogs, cats, rabbits, and guinea-pigs is a cause of very rapid death. In rabbits the average duration of life after this operation is 9 hours; out of 25 animals of this species, only 1 survived 13 hours, and almost all lived 8, 9, or 10 hours only. In adult dogs and cats the average length of life has been 14 hours, and the greatest period has been 17 hours. In newly-born kittens life lasted, in one case 38 hours, in a second 49. After the extirpation of only one of the supra-renal capsules, life lasted 15 or 16 hours, on an average, in rabbits and guinea-pigs, and 27 hours, on an average, in dogs and cats.—*Med. Times and Gazette.*

